# Runt-related transcription factors: from pathogenesis to therapeutic targets in multiple-organ fibrosis

**DOI:** 10.3389/fcell.2025.1528645

**Published:** 2025-04-28

**Authors:** Yuan Feng, Tianshi Mao, Jifei Yi, Na Zhang, Yinying Gu, Huifen Shen, Jie Chen

**Affiliations:** ^1^ Suzhou Wujiang District Hospital of Traditional Chinese Medicine, Suzhou, China; ^2^ Key Laboratory of Chinese Internal Medicine of Ministry of Education and Beijing, Dongzhimen Hospital, Beijing University of Chinese Medicine, Beijing, China; ^3^ Department of Chinese Integrative Medicine Oncology, The First Affiliated Hospital of Anhui Medical University, Hefei, China; ^4^ Department of Integrated Traditional Chinese and Western Medicine, Anhui Medical University, Hefei, China

**Keywords:** fibrosis, Runx, transcription factor, multiple organ, therapeutic targets, extracellular matrix

## Abstract

Fibrosis is a partially manageable process that leads to scarring and tissue hardening by prompting myofibroblasts to deposit significant amounts of extracellular matrix (ECM) following injury. It results in detrimental consequences and pathological characteristics, which hinder the functioning of associated organs and increase mortality rates. Runt-related transcription factors (*RUNX*) are part of a highly conserved family of heterodimer transcription factors, comprising *RUNX1*, *RUNX2*, and *RUNX3*. They are involved in several biological processes and undergo various forms of post-translational modification. *RUNX* regulates multiple targets and pathways to impact fibrosis, indicating promise for clinical application. Therefore, its significance in the fibrosis process should not be disregarded. The review begins with an objective description of the structure, transcriptional mechanism, and biological function of *RUNX1*, *RUNX2*, and *RUNX3*. A subsequent analysis is made of their physiological relationship with heart, lung, kidney, and liver, followed by a focus on the signaling mechanism of *RUNX* in regulating fibrosis of these organs. Furthermore, potential agents or drugs targeting *RUNX* for treating organ fibrosis are summarized, along with an evaluation of the therapeutic prospects and potential value of *RUNX* in fibrosis. Further research into *RUNX* could contribute to the development of novel therapeutic approaches for fibrosis.

## 1 Introduction

The term “fibrosis” was coined in the late 19th century, derived from the Latin word “fibro” meaning fiber, and the Greek/Latin suffix “osis” indicating a pathological condition. Fibrosis is not a disease itself but rather a result of dysfunctional tissue repair in various damaged tissues, particularly during chronic inflammatory diseases (Wen et al., 2022; Nie et al., 2022). It appears in almost all chronic diseases at their end stages, leading to organ dysfunction or failure. Essentially, it involves the progressive accumulation of extracellular matrix (ECM) to repair defects and maintain the integrity of damaged tissues and organs (Huang and Ogawa, 2020; Chen J. et al., 2023). However, restoring the original structure and function of the affected tissue and organ is nearly impossible. Uncontrolled fibrosis threatens tissue survival and function, resulting in organ failure and potentially death. Approximately 45% of disease-related deaths are attributed to fibrosis (Mehal et al., 2011). Fibrosis occurs in nearly all organs, especially vital ones such as the heart, lungs, kidneys, and liver. Moreover, many common diseases, particularly chronic ones, can trigger fibrosis, exacerbating these conditions. For instance, after myocardial infarction (MI), inflammatory cascades aggravate inflammation and activate transforming growth factor-β (TGF-β) signaling, leading to myocardial fibrosis, and eventually heart failure (Zhang Q. et al., 2022). Although the Coronavirus Disease 2019 (COVID-19) outbreak has been effectively controlled worldwide, pulmonary fibrosis remains a challenging complication in recovering patients (Chung et al., 2021). However, the complex pathological mechanisms and dynamic progression of fibrosis render current treatments insufficient. Thus, new strategies are required to restrict the development and progression of multi-organ fibrosis.

Transcription factors play a crucial role in monitoring growth and development in mammals (Weidemüller et al., 2021). Numerous studies suggest that regulating transcription factors can help prevent and treat fibrosis (Liu et al., 2019; Xi et al., 2022; Ma et al., 2023). Runt-related transcription factors (*RUNX*) belong to a family of heterodimeric transcription factors that are widely expressed across various tissues, playing a significant role in numerous biological processes. Previous research has identified the pivotal functions of *RUNX* in craniofacial growth, bone formation, and joint stability. RUNX proteins undergo several post-translational modifications, such as phosphorylation (Guo and Friedman, 2011), acetylation (Jin et al., 2004), ubiquitination (Li et al., 2022), SUMOylation (Kim et al., 2014), and methylation (Zhao et al., 2008), indicating their functional diversity and integrity. Current findings demonstrate that *RUNX* regulates various targets and pathways in fibrosis. Understanding how *RUNX* regulates fibrosis is crucial for uncovering the molecular mechanisms of fibrosis-related ailments and developing effective treatments. This review discusses the role of *RUNX* in the development of multi-organ fibrosis, including in the heart, lungs, kidneys, and liver. It also summarizes potential strategies for managing fibrosis by targeting *RUNX* through pharmacological means.

## 2 Pathomechanism of fibrosis

Fibrosis is a pathological consequence of abnormal organ healing. Despite the varied causes of organ fibrosis, common factors exist. Following injury, increased production of TGF-β, recruitment of inflammatory cells, release of reactive oxygen species, and activation of mesenchymal cells like fibroblasts disrupt the dynamic balance of ECM, leading to excessive deposition of ECM components such as collagen, elastin, laminin, and proteoglycans. Additionally, damaged epithelial cells temporarily lose cell polarity and intercellular junctions, undergo epithelial-mesenchymal transition (EMT), and thus losing their epithelial phenotype while acquiring a mesenchymal phenotype, which in turn activates into myofibroblasts that secrete ECM proteins.

### 2.1 Activation of myofibroblasts

Myofibroblasts are the key cells driving fibrosis. In pathological scarring, myofibroblasts have a heightened sensitivity to signals like chemokines and cytokines, resulting in the overproduction of collagen, fibronectin, and other ECM components, which cause organ fibrosis (Wei et al., 2022). Fibroblasts represent the primary source of myofibroblasts. During the process of fiber formation, cytokines facilitate the proliferation of fibroblasts to migrate towards the wound site and promote their differentiation into myofibroblasts. Epithelial cells from the lungs, liver, and kidneys can undergo EMT under the stimulation of pro-fibrotic factors like TGF-β1, transforming into myofibroblasts. Mesothelial cells stimulated by TGF-β1 or other factors can undergo genetic reprogramming and transform into mesenchymal cells, a process called mesothelial-to-mesenchymal transition, crucial for myofibroblast formation and fibrosis. Myofibroblasts can also originate from endothelial cells and pericytes (Zhao et al., 2022; Weiskirchen et al., 2019). Under pathological conditions, myofibroblasts remain activated by inflammatory cells and fail to be cleared from the wound tissue or organ through normal apoptosis, leading to excessive ECM generation and ultimately resulting in organ fibrosis (Cohen et al., 2023). Targeting myofibroblasts to promote their apoptosis could be a strategy to prevent and treat organ fibrosis in cases of abnormal tissue repair.

### 2.2 Excessive amounts of cytokines

Numerous cytokines, including growth factors and inflammatory factors, play crucial roles in inflammation and fibrosis progression. TGF-β1 is a prominent pro-fibrotic growth factor that recruits inflammatory cells, promotes α-smooth muscle actin (α-SMA)-positive myofibroblast proliferation and differentiation, induces EMT, and enhances the secretion and deposition of collagen and other matrix proteins (Huo et al., 2021). It also suppresses matrix protein degradation by increasing the expression of tissue inhibitors of metalloproteinase (TIMPs), which inhibit matrix metalloproteinases (MMPs), thereby promoting fibrosis. Platelet-derived growth factor activates phosphorylation of phosphoinositide 3-kinase (PI3K)/protein kinase B (AKT) through tyrosine kinase receptors to mediate its biological effects (Fan et al., 2014). This process consequently leads to fibroblast activation, proliferation, migration, and activation of mesenchymal stem/progenitor cells, thus promoting the formation of myofibroblasts, and expediting pulmonary and myocardial fibrosis.

MMPs are the primary enzymes in the breakdown of ECM. Their activity is typically low but increases during tissue repair or remodeling. MMP inhibitors can impede the activity of these enzymes and promote fibrogenesis (Cabral-Pacheco et al., 2020). As a class of pleiotropic cytokines, the interleukins (ILs) are pivotal mediators in diverse inflammatory responses and closely related to the process of fibrosis. *In vivo* studies have demonstrated that fibroblasts treated with IL-1β differentiate into myofibroblasts, leading to ECM deposition. The induction of hepatic fibrosis by IL-13 occurs directly through the upregulation of genes associated with fibrosis, including collagen and connective tissue growth factor. IL-33 promotes hepatic and pulmonary fibrosis by suppressing MMP-9 expression and enhancing TIMP1 expression, thereby creating an imbalance between MMPs and TIMP1 (Zhang et al., 2021; Li, 2011). Soluble IL-33 can produce a fibrotic phenotype in myocardial tissue (Wang et al., 2022a). Elevated IL-38 levels in the peripheral blood of patients with MI, suggest its role in MI and its inhibition of IL-36R signaling in MI progression (Wei et al., 2020). C-C motif chemokine ligand 20 (CCL20) is widely distributed across liver tissues and its over-activation can accelerate hepatic fibrosis by promoting hepatic inflammatory response (Hanson et al., 2019).

Cytokines may act as autocrine or paracrine agents by binding to specific receptors and initiating intracellular signaling pathways that regulate downstream gene expression, ultimately promoting hepatic fibrosis. It is crucial to targeting cytokine biological activity and gene expression for preventing or treating fibrosis.

### 2.3 Dysfunction of signaling pathways

Organ fibrosis entails complex cellular mechanisms, primarily driven by the TGF-β1/Smad pathway. When TGF-β1 binds to the receptor, it phosphorylates Smad2 and Smad3. These activated Smad2 and Smad3 then combine with Smad4 to constitute a trimeric complex, that moves to the nucleus to regulate the transcription of target genes. This complex promotes the proliferation and differentiation of fibroblasts and mesenchymal stromal cells, secretes collagen types I, III, and IV, and induces TIMP1 production. Furthermore, it inhibits MMP-1 production, which prevents ECM degradation, culminating in deposition of ECM and tissue fibrosis (Hu et al., 2018).

Other signaling pathways also play crucial roles in transmitting signals from the cell surface to the nucleus. Members of the mitogen activated protein kinase (MAPK) family, such as p38-MAPK and extracellular signal-regulated kinase 1/2 (ERK1/2), are notable examples. The p38-MAPK signaling pathway, activated by TGF-β1, α-SMA, and other inflammation-related factors triggers the expression of pro-fibrotic genes. The ERK1/2 signaling pathway additionally aids in initiating the Smad pathway via TGF-β1 within RAS/RAF/MAPK/ERK pathway (Dolivo et al., 2021).

The PI3K/AKT signaling pathway, activated by receptor tyrosine kinases, is critical for organ fibrosis development. This pathway regulates mammalian target of rapamycin (mTOR), which controls the survival, proliferation, and differentiation of inflammatory cells. It also modulates cytokine and chemokine expression, promotes ECM remodeling, and ultimately contributes to fibrosis. Downstream proteins, including p70S6K and p-S6, activated by the PI3K/AKT pathway, are crucial for fibrotic disease progression as they promote myofibroblast overproliferation (Dolivo et al., 2021; Jiménez-Uribe et al., 2021). The Hippo/Yes-associated protein (YAP)/transcriptional coactivator with PDZ-binding motif (TAZ) pathway significantly enhances fibroblast proliferation and differentiation of in idiopathic pulmonary fibrosis (IPF) and upregulates fibrosis-related transcriptional genes (Gokey et al., 2021; Fu et al., 2017; Sun et al., 2021). Hedgehog signaling, present in epithelial cells of multiple organs, inhibits E-calmodulin expression and induces α-SMA, Desmin, Snail1, fibronectin, and collagen I production through glioma-associated oncogene one activation (Kumar et al., 2021).

Generally, the process of fibrosis can be divided into five characteristic stages: organ damage, activation of effector cells, production and accumulation of ECM, dynamic deposition of connective tissue, and tissue remodeling. Tissue damage is the precursor to fibrosis, and as the disease progresses, abnormal tissue morphology and organ architecture are disrupted, ultimately leading to organ dysfunction and even failure. The role of transcription factors cannot be ignored in this process. Peroxisome proliferator-activated receptor-γ (PPARγ), as one of the receptors with transcriptional nuclear hormone functions, exhibits a potent antifibrotic effect (Chen H. et al., 2023). It affects ECM synthesis and degradation, myofibroblast morphology, and inhibits the expression of fibrogenic factors. Activated transcription factor 3 responds to TGF-β stimulation and controls the expression of the EMT markers Snail, Slug and Twist to mediate cardiac fibrosis (Hwang et al., 2023). The transcription factor *PU.1* belongs to the E26 transformation-specific family. Its enrichment at the promoter of pro-fibrotic genes in the fibroblast regulatory network suggests its involvement in the activation of fibroblasts (Wohlfahrt et al., 2019).

## 3 Runt related transcription factors

### 3.1 Structural features of the RUNX family

**FIGURE 1:**
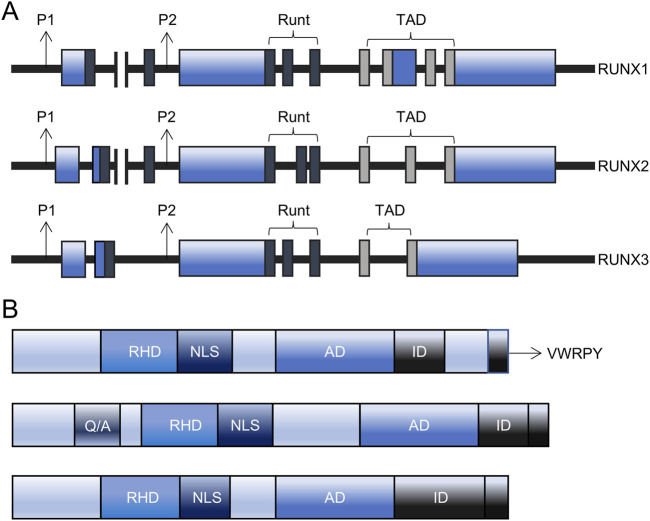
The structure of *RUNX*. **(A)** The structure of the RUNX gene comprises of P1 and P2 promoters, Runt, transactivation domain (TAD), and UTR coding region. **(B)** The structure of the RUNX protein comprises of RHD, AD, ID, NLS, VWRPY, and RUNX2-specific glutamine/alanine-rich region. AD: activation domain, ID: inhibitory domain, NLS: nuclear localization signal, P1: distal promoter, P2: proximal promoter, Q/A: Glutamine/Alanine-rich domain, RHD: Runt homology domain, RUNX: Runt-related transcription factors, TAD: transactivation domain, VWRPY: valine-tryptophan-arginine-proline-tyrosine.

The *RUNX* family comprises three members: *RUNX1*, *RUNX2*, and *RUNX3*. These members exhibit tissue-specific expression patterns and perform specific functions in mammals (Dutta and Osato, 2023). Each *RUNX* member contains a distal promoter (P1) and a proximal promoter (P2), which are activated at different differentiation stages to generate RUNX proteins with distinct N-termini (Figure 1A). The DNA-binding domain (Runt domain) is highly conserved across all RUNX members. The Runt domain, comprising 128 amino acids situated proximal to the N-terminus, is vital for the interaction of the RUNX protein with DNA, other proteins, and nuclear localization. In contrast, the C-termini is less conserved and supports diverse functions through its interaction domains, including transactivation domains (TAD), inhibitory domains (ID), nuclear localization signals (NLS), and valine-tryptophan-arginine-proline-tyrosine (VWRPY) motifs (Figure 1B).

### 3.2 Transcriptional mechanism of RUNX family

**FIGURE 2:**
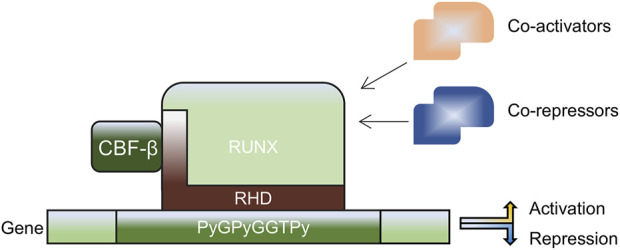
Mechanism of action of the RUNX family. RUNX forms heterodimers with CBF-β through an RHD consensus sequence, enhancing gene transcription when interacting with co-activators, including histone acetyltransferase p300 and cAMP-response element binding protein (CREBBP), and suppressing gene transcription when interacting with co-repressors, including Sin3A, Groucho/transducin-like enhancer protein (TLE) and histone deacetylases. CBF-β: core-binding factor subunit beta, RHD: Runt homology domain, RUNX: Runt-related transcription factors.

*RUNX* transcription factors can both activate and inhibit transcription. The essential binding partner protein, core-binding factor subunit beta (CBF-β), forms heterodimers with the RUNX proteins, resulting in conformational changes that bind to DNA with high affinity, forming stable complexes that either activate or inhibit downstream targets (Mevel et al., 2019). *RUNX* members are considered as weak transcription factors that require interactions with other proteins to enhance their activity (Lee, 2022). Moreover, the three *RUNX* members can functionally compensate for each other. For instance, other *RUNX* members compensate for the antitumor effect of RUNX1 (Lee et al., 2019). Various post-translational modifications regulate the transcriptional efficacy and stability of *RUNX* (Figure 2).

*RUNX* transcription factors can regulate both their own expression and that of each other. In hematopoietic cells, *RUNX1* controls its own expression, while in human B lymphocyte cells, *RUNX3* represses its own expression to regulate *RUNX1* levels (Martinez et al., 2016; Voon et al., 2015). Functional compensation among RUNX members is also observed in leukemia, where other RUNX members compensate for the antitumor effects of *RUNX1* (Morita et al., 2017). However, this compensation is not always consistent. In summary, the roles and interactions of *RUNX* members are complex and possibly subject to the temporal and spatial variables.

### 3.3 Biological functions of RUNX

*RUNX* family members oversee several cellular and developmental processes. The complete knockout of all three *RUNX* members affects survival and highlights their unique functions. *RUNX1* ^−/−^ mice demonstrate vascular defects and fail to establish definitive hematopoiesis during embryogenesis. *RUNX2* ^−/−^ mice display skeletal and cartilaginous malformations. Additionally, *RUNX3* ^−/−^ mice experience impaired lymphangiogenesis and neurogenesis, and suffer from gradually increased gastrointestinal hyperplasia as they age (Mevel et al., 2019; Sweeney et al., 2020). As research on stage- and tissue-specific conditional knockout models progresses, the importance of RUNX proteins is becoming more apparent in hematopoiesis, neurology, immunity, and the skeletal and cardiovascular systems.

The regulatory mechanisms of *RUNX* on genes can be categorized into three main groups: direct regulation of downstream target genes, binding to miRNA and long non-coding RNA (lncRNA) to control critical genes, and interacting with other transcription factors to modulate target genes.

### 3.4 Characteristics of RUNX1, RUNX2, RUNX3

#### 3.4.1 RUNX1

The *RUNX1* gene, located on chromosome 21, was first identified in 1991 and named the acute myeloid leukemia gene due to its involvement in the translocations in patients with acute myeloid leukemia (Wei et al., 2022). *RUNX1*, also known as polyomavirus enhancer-binding protein 2αB subunit or CBF-α2 subunit. Regulates a variety of signaling pathways, including TGF-β, nuclear factor kappa-B (NF-κB), wingless-related integration site (Wnt), and Hedgehog. It serves as a significant downstream factor of the TGF-β pathway, particularly in regulating TGF-β-induced EMT. *RUNX1* promotes EMT by directly interacting with β-catenin and activating KIT transcription to enhance the Wnt signaling pathway. The Wnt/β-catenin pathway, in turn, regulates the downstream localization and expression of *RUNX1* (Li Q. et al., 2019). *RUNX1* also acts as an upstream transcriptional regulator for the actin-related proteins 2/3 complex, influencing downstream molecules like vimentin and thrombospondin-1 thrombin-sensitive protein 1 (Rada et al., 2021; Rada et al., 2022). Furthermore, *RUNX1* may be implicated in inflammation. *RUNX1* binds to p50 (but not p65) and acts as a transcriptional coactivator to synergistically promote IL-6 and IL-1β production in macrophages. The C-terminal fragment of *RUNX1* is responsible for activating NF-κB, containing an activating structural domain (Luo et al., 2016). It is suggested that p50 may cooperate with this structural domain of *RUNX1* to activate gene expression.

#### 3.4.2 RUNX2

*RUNX2* serves as a crucial transcription factor regulating osteoblast maturation and chondrocyte differentiation, playing a significant regulatory role in bone and cartilage metabolism (Komori, 2019). The CBF-β-RUNX heterodimer complex, formed by the binding between *RUNX2* and CBF-β, exhibits higher DNA binding affinity and stability, thereby regulating the expression of various target genes (Wang C. et al., 2021). *RUNX2* also drives EMT heterogeneity through epigenetic mechanisms by binding to the promoters and potential enhancers of EMT-associated genes. Both *in vitro* and *in vivo* genetic experiments have confirmed the EMT-enhancing properties of *RUNX2* (Yan et al., 2020). Furthermore, *RUNX2* facilitates osteoblast differentiation and is linked to bone metabolism, cardiovascular system ectopic calcification, dental development, tumors, and organ fibrosis. Several MMPs, such as MMP-1, 7, 8, 13, 17, 21, and 26, have a promoter site for the binding of RUNX2, allowing RUNX2 to regulate their expression. These MMPs are involved in ECM remodeling, and their dysregulated activity can contribute to fibrosis by promoting excessive ECM turnover and deposition, thus impacting fibrosis pathogenesis (Gonzalez-Avila et al., 2023).

The PI3K/AKT pathway is an intracellular signaling pathway responsible for cell proliferation, apoptosis, angiogenesis, and glucose metabolism (Xie et al., 2019; Keppler-Noreuil et al., 2016). *RUNX2* interacts with this pathway, and its expression and activity are stimulated directly or indirectly through the PI3K/AKT pathway (Cohen-Solal et al., 2015). *RUNX2* also regulates components of the PI3K/AKT pathway or the mTOR complex 2, activating them (Cohen-Solal et al., 2015). Moreover, the activation of ERK-MAPK by basic fibroblast growth factor enhances *RUNX2* transcriptional activity of through *RUNX2* protein phosphorylation, leading to increased intracellular *RUNX2* protein levels (Naderi, 2015).

#### 3.4.3 RUNX3

*RUNX3* is a tumor suppressor gene situated on the 1p36 chromosome in humans. The protein product of RUNX3 is a heterodimer consisting of α and β subunits, comprising 415 amino acid residues and weighing approximately 44 kDa. The protein’s carboxy terminus, rich in serine and proline, binds to DNA to regulate *RUNX3* transcription. DNA methylation involves transferring methyl groups to specific base pairs, catalyzed by DNA methyltransferase and utilizing S-adenosyl methionine as the methyl donor. Excessive consumption of high-methionine foods like eggs or chicken may increase the risk of *RUNX3* methylation (Zhang et al., 2013). *RUNX3* inactivation in cancer can also result from loss of heterozygosity (Mori et al., 2005; Nomoto et al., 2008). In the classical RUNX3/TGF-β pathway, *RUNX3* binds specifically to Smads and activates the p21 promoter. Consequently, the transcription of the pro-apoptotic gene BIML and the expression of the cyclin-dependent kinase inhibitor p21^WAFI^ are enhanced, leading to G1 phase cell arrest and inhibited cell proliferation (Wang et al., 2022b). Additionally, *RUNX3* sequesters β-catenin in the nucleus, inhibiting its role as a transcriptional activator, and interacts with β-catenin/T-cell factor 4 to inactivate Wnt signaling (Zhang et al., 2020).

## 4 The physiological link among RUNX and heart, lung, kidney, liver

### 4.1 Heart

Among the Runt transcription factor family, *RUNX1* is highly expressed in developing embryos. In neonatal hearts, *RUNX1* abundance surpasses that in adult hearts, possibly due to promoter methylation. Genes with *RUNX1* binding sites in the promoter region were found in neonatal rats within 7 days of birth, resulting in a decrease in cardiac proliferation and regeneration (Górnikiewicz et al., 2016). Gene set enrichment analysis of public datasets revealed that *RUNX1*, as a candidate gene for ECM regulation, is mapped to chromosome 21. In human fetal fibroblasts with chromosome 21 trisomy, approximately 80% of ECM genes exhibit a consistent *RUNX1* sequence in the promoter (Mollo et al., 2022). The gene dosage effect caused by the overexpression of *RUNX1* leads to the upregulation of ECM genes, which in turn alters the cardiac morphology and function during development. Moreover, *RUNX2* contributes to the formation of cardiac valves during embryonic development. During successive rounds of EMT and mesenchymal-epithelial transition, *RUNX2* acts as a downstream transcription factor for the formation of atrioventricular and ventricular outflow tract valves. In embryonic mouse hearts, *RUNX2* is present in both endocardial cells and mesenchyme throughout mesenchymal transformation. Additionally, substrate stiffness regulates the proliferation of cardiac fibroblasts (Wang X. et al., 2021), while changes in actin cytoskeleton organization activate YAP. Matrix stiffness regulates the activities of *RUNX2* and transcriptional enhanced associate domain in cardiac fibroblasts through YAP. YAP-RUNX2 and YAP-transcriptional enhanced associate domain pathways work together to promote the proliferation of cardiac fibroblasts on stiff matrices (Ebrahimighaei et al., 2022). *RUNX3* engages in function of cardiac microvascular endothelial cells. It maintains the phenotype of mesenchymal cells transformed by Notch signaling during cardiac development and regulates EMT in these cells (Piera-Velazquez and Jimenez, 2019).

### 4.2 Lung

Cellular proliferation, differentiation, and EMT are essential for pulmonary development. *RUNX1* is highly expressed in bronchial epithelial cells in both mice and human lung tissue (Levanon et al., 2001). Genetic variations that altered *RUNX2* binding in the secreted phosphoprotein 1 (Spp1) promoter, along with promoter variations leading to increased DNA protein binding at the *RUNX2* binding site, were identified in JF1/Msf mice with reduced lung function (Ganguly et al., 2014). *RUNX2* binding to *Spp1* may be a determinant of pulmonary function development. At postnatal day 1, *RUNX3* ^−/−^ mice exhibited abnormal alveolar remodeling compared to wild-type mice, and their bronchiolar epithelial cells showed reduced differentiation capacity (Lee et al., 2010). The absence of *RUNX3* in mice led to elevated levels of inducible factors, such as Smad 2, 3, 4, Slug, Snail and TGF-β1 (Lee et al., 2011). This suggests an irregular EMT process leading to abnormal alveolar development in *RUNX3* ^−/−^ mice. Thus, *RUNX3* may help prevent abnormal EMT and maintain healthy pulmonary development by regulating specific genes.

### 4.3 Kidney

Hematopoietic stem cells (HSCs) are believed to originate from the hemogenic endothelium in the dorsal aorta, migrating later to the fetal liver and bone marrow in mammals. A recent single-cell transcriptomics of cortex tissues from two human fetal kidneys confirmed the presence of the HSCs and detected the *RUNX1* gene in human in *ex vivo* human fetal kidney cortex (Hwang et al., 2021). Although the kidney is crucial for HSC maintenance, their maintenance and proliferation are primarily regulated within their ecological niche in the bone marrow. An inhibitor, which specifically inhibits the canonical Wnt signaling pathway, effectively regulates HSCs in the zebrafish kidney stroma by maintaining but not expanding them, acting through *RUNX1* (Kimura et al., 2022). SPI1 (also known as PU.1) serves as a crucial downstream target of *RUNX1,* inhibiting excessive proliferation and preventing the exhaustion of HSCs by regulating various cell cycle regulators (Imperato et al., 2015; Staber et al., 2013). The interaction between SPI1 and *RUNX1* can transform hematopoietic endothelium into hematopoietic stem and progenitor cells, leading to the development of myeloid, B, and T cells in both primary and secondary mouse recipients (Sugimura et al., 2017). *RUNX1* integrates the GATA, ETS, and SCL transcriptional networks, along with cis-regulatory elements, playing a significant role in HSC generation (Nottingham et al., 2007). *RUNX2* expressed in the tubular epithelial cells of both mouse and human kidneys (Kramann and Schneider, 2013) and enhances fibronectin one protein expression through Calpain-2 (Zhang X. et al., 2022). A reduction in *RUNX3* expression may indicate a poor prognosis and contribute to tumor progression in kidney cancer (Zheng et al., 2020).

### 4.4 Liver

Appropriate *RUNX* levels are crucial for liver development. Angiogenesis, a vital aspect of embryonic organogenesis and tissue repair, is facilitated by HSCs during embryonic development (Hsu et al., 2000). *RUNX1* deficiency in embryos leads to poor hematopoiesis and defective liver angiogenesis (Suda and Takakura, 2001). Additionally, HSCs can differentiate into diverse mature cell types, including non-hematopoietic lineages, and populate the liver. During this differentiation, *RUNX1* plays a fundamental role by interacting with multifactor complexes to activate tissue-specific and synergistic genes (Saunthararajah et al., 2006).

## 5 RUNX is involved in multiorgan fibrosis

### 5.1 Cardiac fibrosis

Cardiac fibrosis, a common pathological change in various heart conditions, primarily characterizes myocardial structural remodeling. It is an important cause of arrhythmia, sudden cardiac death, and chronic heart failure. There are two main types of myocardial fibrosis: replacement fibrosis and diffuse fibrosis. Replacement fibrosis, also referred to as scar fibrosis, mainly results from the apoptosis of cardiomyocytes. When myocardial cells undergo apoptosis caused by acute injury, dormant fibroblasts become activated, proliferating and differentiating into myofibroblasts that repair the scar. In contrast, diffuse myocardial fibrosis involves a widespread increase in extracellular collagen, also known as interstitial fibrosis. Unlike replacement fibrosis, diffuse fibrosis rarely causes apoptosis of myocytes and is usually due to prolonged cardiac stress and abnormalities in signaling molecules.

RNA-seq analysis of acute MI rat models revealed that *RUNX1* was the gene with the highest differential expression related to MI (GSE46395). As early as 1 day after MI, *RUNX1* expression increases in cardiomyocytes in the border zone and infarct zone, which impairs Ca^2+^ handling, reducing mitochondrial density and oxidative phosphorylation gene expression, thereby reducing myocardial contractility (Martin et al., 2023). *RUNX1* has been implicated in negative cardiac remodeling mechanisms (McCarroll et al., 2018). Its activation in cardiomyocytes post-MI has a detrimental effect on ventricular function. Reducing *RUNX1* levels decreased α-SMA expression and increased the expression of punctate gap junction protein connexin 43 (CX43), a critical intercellular communication channel regulating cardiomyocyte-fibroblast crosstalk (Ni et al., 2021). Single cell sequencing results from zebrafish hearts supported the role of *RUNX1* as an inhibitor of cardiac repair on various levels. Injured cardiac sites contained *RUNX1*-positive endocardial cells and thrombocytes, which promoted the expression of genes promoting smooth muscle and collagen deposition but hindered myocardial regeneration. In contrast, cardiac injuries in zebrafish with *RUNX1* mutations displayed decreased collagen and fibrin levels, suggesting that targeting *RUNX1* may offer a groundbreaking therapeutic approach for enhancing natural heart repair (Koth et al., 2020). miR-101 directly targets and represses *RUNX1* expression, reducing TGF-β1/Smad 2 signaling activity. This results in a decrease in myocardial fibrosis and cardiomyocyte apoptosis (Li X. et al., 2019). Myocardial fibrosis is the primary manifestation of cardiomyocyte dedifferentiation, which, if prolonged, can lead to heart failure. *RUNX1* serves as a common marker for this dedifferentiation. Oncostatin M (OSM), an inflammatory cytokine from the IL-6 family of cytokines, is recognized as a pro-fibrotic mediator in the heart, capable of stimulating cardiac fibroblast proliferation, ECM production, and inflammatory responses, thus promoting cardiac fibrosis. In diabetic cardiomyopathy mice, the depletion of OSM can reduce the fibrosis area by abrogating the B-Raf/MEK/ERK signaling pathway and decreasing RUNX1 levels (Zhang et al., 2016).

**FIGURE 3:**
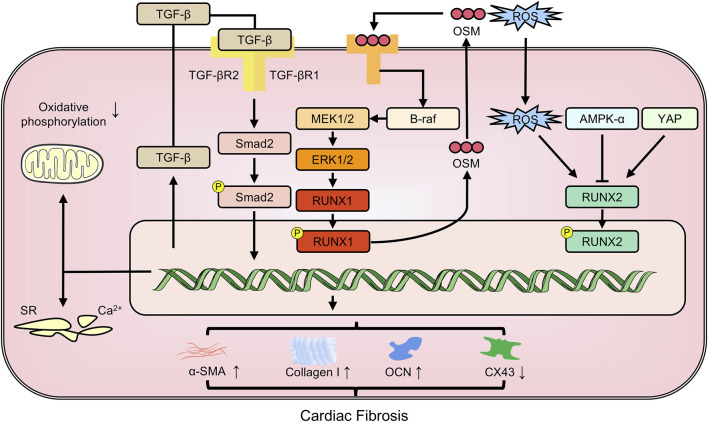
The members of RUNX family regulate cardiac fibrosis involving in multiple pathways. CX43: connexin43; OCN: osteocalcin; OSM: oncostatin M; YAP: yes-associated protein; α-SMA: alpha-smooth muscle actin.

The activation and proliferation of resident cardiac progenitor cells post-MI are critical for endogenous cardiac regeneration and repair. These seemingly divergent effects may stem from differences in the spatio-temporal expression of *RUNX1*. Beyond myocardial fibrosis, it is possible that the *RUNX* family might also contribute to pericardial fibrocalcification. *RUNX2*, as a transcription factor essential for osteoblast differentiation, drives pericardial interstitial cells and myofibroblasts to osteoblasts to form pericardial fibrosis (Liu et al., 2012). In type 2 diabetic mice, the specific overexpression of *RUNX2* in vascular smooth muscle cells increased its target genes, Col1a1 and Col1a2, causing medial aortic fibrosis and aortic sclerosis (Raaz et al., 2015). Additionally, *Klotho* deficiency may exacerbate high fat diet-induced aortic valve fibrosis caused by upregulation of matrix proteins including collagen I and osteocalcin (OCN, a bone-derived hormone promoting vascular calcification) *via* the adenosine 5‘-monophosphate-activated protein kinase α-RUNX2 pathway (Chen et al., 2016) (Figure 3).

### 5.2 Pulmonary fibrosis

Pulmonary fibrosis is a late-stage modification of respiratory diseases, characterized by pulmonary dysfunction and respiratory failure as a consequence. This condition can result from coronavirus infections including severe acute respiratory syndrome and the Middle East respiratory syndrome, and COVID-19 (George et al., 2020). Exposure to fine particulate matter, particularly diesel exhaust PM2.5, increases the risk and accelerates the progression of pulmonary fibrosis (Yue et al., 2023). Of particular clinical significance, IPF represents a distinct and severe form of chronic progressive fibrosing interstitial pneumonia of unknown etiology, predominantly affecting older adults. IPF is characterized histopathologically by usual interstitial pneumonia patterns featuring temporal heterogeneity, fibroblast foci, and honeycombing changes (Moss et al., 2022). Notably, current anti-fibrotic therapies for IPF (nintedanib and pirfenidone) demonstrate partial efficacy by slowing decline rather than reversing established fibrosis, underscoring the need for better understanding of underlying mechanisms. The root cause of this disease is the abnormal architecture of lung tissue, arising from improper repair of damaged alveolar tissue. The main mechanisms driving pulmonary fibrosis include EMT, fibroblast heterogeneity and plasticity, collagen deposition of ECM, the macrophage polarization and activation, abnormal repair of damaged pulmonary tissue, and cellular senescence. Inflammatory lung diseases characterized by alveolar and diffuse parenchymal inflammation and interstitial fibrosis are collectively known as interstitial lung disease (ILD) (Chen, 2023), with IPF constituting one of the most prognostically dire subtypes. Emerging evidence suggests that RUNX transcription factors may participate in IPF pathogenesis through dysregulation of epithelial-mesenchymal crosstalk and sustained fibroblast activation, though this warrants further investigation.

**FIGURE 4:**
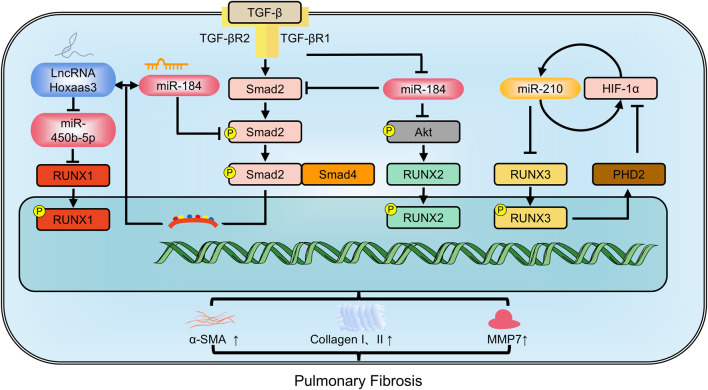
The members of RUNX family regulate pulmonary fibrosis involving in multiple pathways. MMP9: Matrix metalloproteinase-9; PHD2: prolyl hydroxylase domain-containing protein 2; α-SMA: alpha-smooth muscle actin.

*RUNX1* is an upstream transcription factor driving myofibroblast differentiation (Tabib et al., 2021). Upon TGF-β stimulation, *RUNX1* expression increases significantly in the nucleus, due to enhanced mRNA stability facilitated by interaction with the RNA-binding profibrotic protein, human antigen R. Inhibiting *RUNX1* significantly decreases fibroblast differentiation into myofibroblasts (Dubey et al., 2022). LncRNAs, transcripts longer than 200 nucleotides, are also implicated in pulmonary fibrosis (Yao and Lou, 2023). LncRNAs and miRNAs can mutually regulate each other. For instance, the lncRNA Hoxaas3 regulates lung fibroblast activation and fibrogenesis by acting as a competitive endogenous RNA for miR-450b-5p. This reduces miR-450b-5p expression, thereby enhancing *RUNX1* levels and activity, ultimately contributing to fibrosis. Conversely, inhibiting *RUNX1* mitigates the pro-fibrotic effects of Hoxaas3 (Lin et al., 2020). In patients with IPF and in experimental bleomycin-induced pulmonary fibrosis, there is elevated expression of *RUNX2* (Mümmler et al., 2018). *RUNX2* not only reduces diffusion capacity but also increases levels of the IPF biomarker MMP7, correlating with disease severity. Nuclear *RUNX2* is found in hyperplastic epithelial cells positive for prosurfactant protein C, but is rare in myofibroblasts. Knocking down miR-31 and/or an increasing miR-184 suppresses the TGF-β/Smad2 and TGF-β/PI3K/AKT signaling pathways, respectively, thereby inhibiting the expression of pro-fibrotic factors, MMP7, and RUNX2 (Wang et al., 2020). Targeting *RUNX2* pathways in a cell-specific manner may hold promise as a therapeutic approach for pulmonary fibrosis. Conversely, inhibition of *RUNX3* worsens irreversible pulmonary fibrosis induced by paraquat. *RUNX3* may enhance the hydroxylation capacity of prolyl hydroxylase domain-containing protein 2 (PHD2), promote the degradation of hypoxia inducible factor-1α (HIF-1α), and weaken paraquat-induced EMT, thereby partially reversing pulmonary fibrosis (Zhu et al., 2017) (Figure 4).

### 5.3 Renal fibrosis

Renal fibrosis commonly occurs as chronic kidney disease progresses to end-stage renal disease and is its primary histopathological manifestation of this disease. It is a necessary process in various kidney diseases such as chronic nephritis, diabetic nephropathy, hypertensive nephropathy, polycystic kidney disease, and interstitial kidney disease. Renal fibrosis encompasses glomerulosclerosis and interstitial fibrosis, which damage the renal parenchyma and interstitial tissue. The initiation of renal fibrosis necessitates inflammatory cell infiltration. Inflammatory conditions activate and proliferate myofibroblasts from various origins, leading to the accumulation and continuous production of ECM proteins. This results in atrophic renal tubular cells and sparse microvessels. Even the activation of renal tubular cells alone can induce renal fibrosis (Sugimoto et al., 2012). A hallmark of pathological renal fibrosis is the altered gene expression patterns in renal epithelial cells, known as partial EMT (Galichon et al., 2013).

**FIGURE 5:**
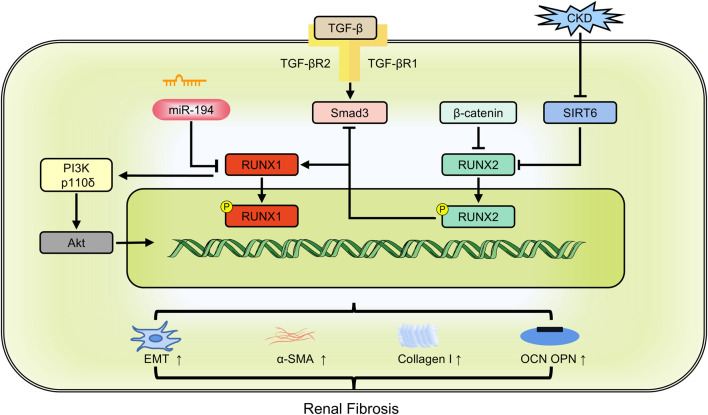
The members of RUNX family regulate renal fibrosis involving in multiple pathways. CKD: chronic kidney disease; EMT: epithelial-mesenchymal transition; OCN: osteocalcin; OPN: osteopontin; α-SMA: alpha-smooth muscle actin.

*RUNX1* is a significant aberrant transcription factor in polycystic kidney disease and kidney injury (Formica et al., 2019). Its expression is elevated in the renal tubules of two distinct models of chronic kidney disease models: unilateral ureteral obstruction and folic acid treatment. *RUNX1* may promote TGF-β-induced renal tubular EMT and fibrosis by upregulating PI3K subunit p110δ transcription. Specific deletion of *RUNX1* mitigated both TGF-β-induced phenotypic changes and renal fibrosis. Although Snail1 expression occurs before *RUNX1* induction, it depends on *RUNX1* for signaling transduction, underscoring the role of *RUNX1* as a fibrosis switch (Zhou et al., 2018). miR-194 acts as an upstream regulator of *RUNX1*. Overexpressing miR-194 hindered α-SMA and collagen I expression, thereby lessening renal fibrosis through the inhibition of the RUNX1/AKT pathway (Cheng et al., 2020). Single-cell ATAC sequencing data further identified *RUNX1* and its target genes in promoting fibroblast to myofibroblast differentiation, driving renal fibrosis (Li et al., 2021). TAFRO syndrome, a subtype of idiopathic multicentric Castleman disease, is clinically characterized by systemic inflammation (Fajgenbaum et al., 2020). Patients with TAFRO syndrome exhibit renal fibrosis and dysfunction. *RUNX1* mutations in these patients disrupt *RUNX1* transcriptional activity, leading to dominant negative *RUNX1* and increased self-renewal of hematopoietic stem/progenitor cells (Yoshimi et al., 2020). *RUNX2* affects TGF-β signaling. In mouse embryonic fibroblasts, *RUNX2* deficiency induced Smad3 activation and enhanced α-SMA expression in the presence of TGF-β. Conversely, *RUNX2* overexpression reduced TGF-β-induced Smad3 phosphorylation, thereby decreasing α-SMA and collagen I expression (Kim et al., 2013). *RUNX3* could be recognized as a predictor of risk for progressive interstitial fibrosis and tubular atrophy (Sigdel et al., 2015) (Figure 5).

### 5.4 Hepatic fibrosis

Hepatic fibrosis is an early pathological change that occurs as the liver attempts to repair itself after injury from various causes, including viral hepatitis, cholestasis, fatty liver, etc. It arises from a complex multicellular response where the liver engages in self-repair and wound healing. Chronic or persistent liver injury primarily results in hepatic fibrosis, which can progress to life-threatening cirrhosis and hepatocellular carcinoma in advanced stages.

Upon hepatic injury, cell death and immune cell infiltration initiate a cascade of inflammatory and fibrotic signals. Injured cells release danger-associated molecular patterns that activate inflammatory cells, including macrophages, T cells, natural killer cells, and Kupffer cells. These cells secrete pro-inflammatory factors, like IL-1β, IL-18, IL-13, interferon γ, tumor necrosis factor-α (TNF-α), and C-C motif chemokine ligand 2, promoting further recruitment and activation of lymphocytes and monocytes at the injury site. Within this inflammatory microenvironment, hepatic stellate cells (HPSCs) become activated, proliferating and differentiating into myofibroblasts. This leads to excessive ECM accumulation in the liver and gradual fibrous scarring. Endogenous portal fibroblasts, hepatic parenchymal cells, bone marrow-derived cells, and HPSCs contribute to myofibroblasts production through EMT. These cells also produce ECM proteins, such as glycoproteins, proteoglycans, laminin, and fibronectin, thereby influencing hepatic fibrosis.

Alcoholic and nonalcoholic steatohepatitis (NASH) are leading risk factors for hepatic fibrosis. Differences in the expression and function of *RUNX* members are significant in this context. There are significant differences in expression and function among RUNX members. NASH patients exhibit increased expression of *RUNX3*, whereas non-NASH patients have significantly lower expression (Jia et al., 2019). *RUNX1* drives the plasticity of HPSCs associated with nonalcoholic fatty liver (Marcher et al., 2019). Single-cell data from fibrotic/cirrhotic human livers demonstrate the responsibility of *RUNX1* in activating HPSCs during fibrogenesis (Wang Z. Y. et al., 2021). RUNX1 levels correlate strongly with inflammation, fibrosis, and NASH activity scores in NASH patients (Kaur et al., 2019). RUNX1 may also regulate proteins such as NF-κB1, NF-κB2, TNF, ADIPOQ, and IL-6, directly contributing to liver injury (Kaur et al., 2019). Protein ubiquitination and deubiquitination are key modes of protein post-translational regulation. Ubiquitin-specific protease 9X, a deubiquitinating enzyme, removes protein ubiquitination and stabilizes Smad1 expression (Cheng et al., 2012). *RUNX1* can transcriptionally activate the expression of ubiquitin-specific protease 9X and regulate Smad1 expression. Depletion of *RUNX1* decreases the viability and migration of HSPCs, thereby alleviating carbon tetrachloride-induced hepatic fibrosis (Huang et al., 2021).

**FIGURE 6:**
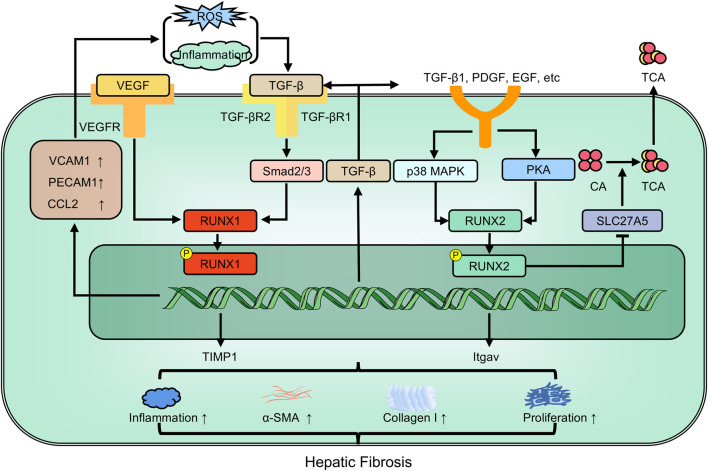
The members of RUNX family regulate hepatic fibrosis involving in multiple pathways. EGF: epidermal growth factor; Itgav: integrin alpha-V; PDGF: platelet-derived growth factor; TIMP-1: tissue inhibitor of metalloprotease-1; α-SMA: alpha-smooth muscle actin.

TIMP1 promotes liver fibrosis (Cong et al., 2009). The UTE-1 regulatory DNA motif is essential for TIMP1 promoter activity in HPSCs. *RUNX* proteins bind to UTE-1 and are upregulated post-transcriptionally during HPSC activation, However, *RUNX* family members are diversely oriented. Overexpression of the full-length *RUNX1B* isoform inhibits TIMP1 promoter activity, whereas the truncated *RUNX1A* isoform and *RUNX2* stimulate it (Bertrand-Philippe et al., 2004). *RUNX2* is also a crucial downstream regulator of the p38-MAPK pathway. Inhibiting p38-MAPK could downregulate RUNX2 levels, thus attenuating hepatic fibrosis (Hattori et al., 2007). Moreover, TGF-β1 targets *RUNX3 in vitro*, leading to increased cell proliferation and differentiation, and resulting in overexpression of miR-130a and miR130b during hepatic fibrosis (Lu et al., 2015). Following TGF-β1 stimulation, *RUNX2* acts on the ACCACA sequence in the osteopontin (OPN) promoter region, activating OPN transcription in human HPSCs and promoting fibrosis. Further studies have indicated that *RUNX2* expression in hepatic cirrhosis tissues positively correlates with disease severity (Wang, 2013) (Figure 6).

## 6 Therapeutic candidate drugs to regulate *RUNX*

Treating fibrosis focuses on managing the primary disease or minimizing tissue damage. Early and effective treatment of the root cause can significantly enhance or even reverse fibrosis.

### 6.1 Chemical agents

FR-167653, a selective p38-MAPK inhibitor, has shown effectiveness in treating cirrhotic rats induced by carbon tetrachloride. It targets p38 and significantly reduces *RUNX2*, thus ameliorating fibrotic changes in HPSCs and portal fibroblasts (Hattori et al., 2007). *RUNX1* siRNA delivery to the lungs *via* micelles formed by a graft copolymer containing multiple polyethylene glycol (PEG) modified branched polyethylenimine could inhibit myofibroblast differentiation in lung-resident mesenchymal stem cells. This offers a potential treatment for pulmonary fibrosis (Ji et al., 2021). Ro24-7429, a safe *RUNX1* inhibitor, has been shown to reduce the downstream pathological mediators of fibrosis (TGF-β1) and inflammation (TNF-α) in cultured lung epithelial cells, fibroblasts and vascular endothelial cells, indicating multiple beneficial effects (O'Hare et al., 2021). The cannabinoid receptor type 2 agonist AM1241 can enhance *RUNX1* expression in cardiac progenitor cells, improve cardiomyocyte proliferation, and reduce fibrosis, thus preserving cardiac function in ischemic myocardium (Wang et al., 2014). The small molecule benzodiazepine Ro5-3335 specifically inhibits RUNX1 activity. It has been shown to improve pulmonary hypertension, retinal angiogenesis, and acute myeloid leukemia by altering vascular remodeling, pulmonary macrophage activity, and endothelial to haemopoietic transition (Jeong et al., 2022; Lam et al., 2017; Richter et al., 2019). A recent study found that Ro5-3335 improves cardiac contractile function after MI, suggesting that it could prevent adverse remodeling after cardiac injury. Gramine, a natural indole alkaloid from the Asian giant reed, can suppress the TGFBR1-p38 MAPK signaling axis by binding to *RUNX1*, thereby ameliorating cardiac hypertrophy and fibrosis (Xu et al., 2023).

### 6.2 Traditional Chinese medicines

Besides chemical drugs, traditional Chinese medicines (TCM) and lead compounds from Chinese herbs may offer potential avenues for developing drugs to treat fibrosis. Dihydrocorine, an isoquinoline alkaloid produced by hydrogenating lycorine extracted from *Lycoris* in the *Lycoraceae* family, acts as a promising *RUNX1* inhibitor. This compound directly downregulates *RUNX1* post-MI, suppressing TGF-β/Smad3, which in turn reduces collagen I and α-SMA, thus alleviating cardiac fibrosis (McCarroll et al., 2018). Bushen Huoxue Decoction is composed of Cuscutae Semen, Taxilli Herba, Dipsaci Radix, Codonopsis Radix, Atractylodis Macrocephalae Rhizoma, Angelicae Sinensis Radix, Salviae Miltiorrhizae Radix, and Chuanxiong Rhizoma. This decoction can inactivate the BMP-2/RUNX2/Osterix signal pathway, thereby alleviating inflammatory infiltration, adenine crystal deposition, renal interstitial fibrosis, and calcified nodes in the renal aorta in chronic renal failure rats (Liu et al., 2016), which emphasizes the applicability of traditional Chinese medicine in contemporary therapy and the vital role of RUNX2.

Chemical drugs stand out for their specificity in targeting defined molecular pathways with precision. This specificity enables them to rapidly alleviate symptoms, essential for acute or severe fibrotic conditions. The adjustable dosage of chemical drugs enables tailored treatment regimens, optimized by well-established pharmacokinetic profiles. Rigorous clinical validation and strict regulatory oversight ensure high confidence in the efficacy and safety of chemical drugs. However, chemical drugs come with notable drawbacks, including the risk of drug resistance, especially with long-term use. Individual variability in drug response among individuals also poses challenges. TCM offers a holistic approach, emphasizing restoring balance within the body. This approach aligns with the growing appreciation for the multifactorial nature of fibrosis, addressing not only the fibrotic processes but also the overall wellbeing of the patient. However, the complex composition of TCM formulations complicates standardization and quality control, making consistent therapeutic outcomes challenging.

The dichotomy between chemical drugs and TCM in treating fibrosis underscores the potential benefits of an integrated treatment model. By leveraging the rapid symptom relief and specificity of chemical drugs alongside the holistic, patient-centered approach of TCM, it may be possible to devise treatment protocols that offer enhanced efficacy, improved patient tolerance, and reduced long-term treatment costs. Going forward, rigorous comparative studies and clinical trials are essential to validate the synergistic potential and optimal integration strategies for integrating chemical drugs and TCM.

## 7 Conclusions and perspectives

Fibrosis is a widespread pathological process that can adversely affect any organ in many diseases. It typically occurs due to the loss of parenchymal cells and the proliferation of connective tissue in organs and tissues, leading to progressive and prolonged structural damage and dysfunction (Wynn and Ramalingam, 2012; Zhao et al., 2020). Although some progress has been made in comprehending fibrosis across multiple organs, the exact mechanisms driving it from different causes still remain unclear. The inability to fully comprehend the mechanisms behind fibrosis has impeded the advancement of efficacious therapies. Nevertheless, since fibrosis shares a common pathological basis across organs, identifying common targets is highly valuable for translation into therapies.

The TGF-β signaling pathway is extensively studied for its role in inducing fibrosis. Although various transcription factors can collaborate with the R-Smad proteins specifically activated by the TGF-β family (Smad2/3, Smad1/5/8), the *RUNX* family is one of its specific targets (Naderi, 2015). *RUNX* family members are more closely linked to the TGF-β superfamily signaling pathway. In recent decades, considerable insights have emerged about the role of *RUNX* in multiple disease lineages and development, particularly in cell function and fate. The *RUNX* family, as specific transcription factors for cell differentiation, has received significant attention in various fields, including bone metabolism, ectopic calcification of the cardiovascular system, tooth development, tumors, and particularly, organ fibrosis. The related molecular pathways have become a subject of intense research in medicine. Further exploration is necessary to fully comprehend how *RUNX* regulates diseases. Although the various members of the *RUNX* family may seem independent, their biological functions and regulatory mechanisms likely resemble complex networks that exhibit overlapping and synergistic functions and mechanisms with constraints. *RUNX1* and *RUNX2* both bind to UTE-1. Different isoforms of the same *RUNX* family member may have diametrically opposite functions. *RUNX1A* directly interacts with JunD to promote the function of TIMP1 transcription, whereas *RUNX1B* does not interact with JunD to inhibit JUN-stimulated TIMP1 promoter activity (Bertrand-Philippe et al., 2004). Meanwhile, embers of *RUNX* family can act together in multiple fibrotic diseases (Roberts et al., 2010). Emerging evidence suggests RUNX1 may contribute to myocardial, renal, and hepatic fibrosis through its regulatory influence on multiple signaling pathways. *RUNX1* and *RUNX2* may work together in fibrosis progression. *RUNX1* is mainly expressed in CXC chemokine ligand 12-abundant reticular (CAR) cells. The absence of both *RUNX1* and *RUNX2* in CAR cells in mice increases fibrosis and bone formation in the bone marrow coupled with a significant decrease in HSCs and progenitors (Omatsu et al., 2022). Given the overlapping functions and cross-regulatory capacity between *RUNX* genes, it is of utmost importance to examine their expression and action patterns in different fibrotic tissues.

On one hand, the study of *RUNX* and its regulation of the pathogenesis of multiorgan fibrosis can help to better understand the association between the course of fibrotic diseases and organ-specific fibrosis, and may enable early screening for fibrotic risk factors; on the other hand, exploring the precise targets of *RUNX*-regulated diseases and searching for relevant targeted therapeutic agents. Exploring natural products may offer a vast reservoir of potential medications. In summary, the *RUNX* family may represent a novel target for treating multi-organ fibrosis in the future.

## Glossary

AKT: protein kinase B
CAR: CXC chemokine ligand 12-abundant reticular
CBF-β: core-binding factor subunit beta
CCL20: C-C motif chemokine ligand 20
COVID-19: coronavirus disease 2019
CX43: connexin 43
ECM: extracellular matrix
EMT: epithelial-mesenchymal transition
ERK1/2: extracellular signal-regulated kinase 1/2
HIF-1α: hypoxia inducible factor-1α
HPSCs: hepatic stellate cells
HSCs: Hematopoietic stem cells
ID: inhibitory domains
IL: interleukin
ILD: interstitial lung disease
IPF: idiopathic pulmonary fibrosis
lncRNA: long non-coding RNA
MAPK: mitogen activated protein kinase
MI: myocardial infarction
MMP: matrix-metalloproteinases
mTOR: mammalian target of rapamycin
NASH: nonalcoholic steatohepatitis
NF-κB: nuclear factor kappa-B
NLS: nuclear localization signal
OCN: osteocalcin
OPN: osteopontin
OSM: oncostatin M
PEG: polyethylene glycol
PHD2: prolyl hydroxylase domain-containing protein 2
PI3K: phosphoinositide 3-kinase
PPARγ: peroxisome proliferator-activated receptor-γ
*RUNX*: Runt-related transcription factors
Spp1: secreted phosphoprotein one
TAD: transactivation domains
TAZ: transcriptional coactivator with PDZ-binding motif
TCM: traditional Chinese medicine
TGF-β: transforming growth factor-β
TNF-α: tumor necrosis factor-α
TIMP: tissue inhibitors of metal proteinase
VWRPY: valine-tryptophan-arginine-proline-tyrosine
Wnt: wingless-related integration site
YAP: Yes-associated protein
α-SMA: α-smooth muscle actin
